# Hemoglobin A1c Screening Practices and Characteristics in Youth with Congenital Heart Disease

**DOI:** 10.1007/s00246-025-04092-0

**Published:** 2025-11-06

**Authors:** Jonathan A. Wheeler, Phillip S. Cheng, Chance Alvarado, Leena Mamilly, Aaron Walsh, Kan N. Hor, Andrew H. Tran

**Affiliations:** 1 Division of Pediatric Cardiology, Department of Pediatrics, Baylor College of Medicine, Texas Children’s Hospital, Houston, TX USA; 2 Department of Pediatrics, The Ohio State University, Columbus, OH USA; 3 The Heart Center, Nationwide Children’s Hospital, 700 Childrens Drive, Columbus, OH 43205 USA; 4 Biostatistics Resource, Abigail Wexner Research Institute, Nationwide Children’s Hospital, Columbus, OH 43205 USA; 5 Center for Biostatistics, Wexner Medical Center, The Ohio State University, Columbus, OH 43210 USA; 6 Section of Endocrinology, Department of Pediatrics, Nationwide Children’s Hospital/The Ohio State University College of Medicine, Columbus, OH USA; 7 The Heart Institute, Le Bonheur Children’s Hospital, Memphis, TN USA

**Keywords:** Pediatric cardiology, Diabetes mellitus, Preventive cardiology, Hemoglobin A1c, Metabolic syndrome, Children

## Abstract

Pediatric congenital heart disease (CHD) patients have increased risk for cardiovascular (CV) disease. Acquired conditions such as elevated Hemoglobin A1c (HbA1c) are known risk factors for adverse CV outcomes in the adult population. With the increasing rates of pediatric prediabetes and diabetes, data are limited on the screening practices for HbA1c in children with CHD. Presence of elevated HbA1c in this population would potentially increase future CV risk. Our study aimed to evaluate screening practices for HbA1c in youth with CHD and the characteristics of those screened. Youth with CHD with primary cardiology encounters from 2012 to 2019 were evaluated. Available anthropometric, demographic, and lab data were analyzed to determine rates of HbA1c screening, screening eligibility, and compare those screened with HbA1c versus those unscreened. Of 2764 total patients, 7.9% (219/2764) had HbA1c labs with 21.9% (48/219) in the prediabetic/diabetic range. Screened patients had a higher proportion of individuals with elevated or hypertensive blood pressure measurements and overweight/obese body mass index versus unscreened patients. Although 213 patients (7.7%) were screen-eligible for HbA1c per American Diabetes Association recommendations, only 27.2% (58/213) were screened with 37.9% (22/58) in the prediabetic/diabetic range. Only a small percentage of screen-eligible patients were screened with HbA1c. Of those screened, a sizable proportion of patients were in the prediabetic/diabetic range. Improved screening practices for HbA1c and metabolic syndrome in children with CHD are needed.

**Supplementary Information:**

The online version contains supplementary material available at https://doi.org/10.1007/s00246-025-04092-0.

## Introduction

Since 1988, national prevalence rates of overweight or obese children and adolescents have increased substantially [1, 2]. Mirroring obesity trends, there has been a similar increase in prevalence and incidence of Type 1 and Type 2 Diabetes [3–5]. The American Diabetes Association (ADA) released guidelines regarding screening of overweight and obese children with additional risk factors including maternal history of diabetes, family history, race, and evidence of insulin resistance [6]. Based on screening guidelines, in 2016 there were 10.6 million children and adolescents, around one-quarter of the US population in this age group, eligible for diabetes screening [7]. In this population who was screening eligible, ~ 4% had Hemoglobin A1c (HbA1c) defined hyperglycemia (≥ 5.7%) [7]. While there are other definitions of diagnosis for prediabetes and diabetes such as fasting plasma glucose [8], cardiometabolic risk factor associations were strongest for those with HbA1c defined hyperglycemia [7].

Congenital heart disease (CHD) is a structural defect of the heart or major cardiac arteries/veins that is present at birth. Patients with CHD are an ever-expanding population with estimates as high as 1 in 150 adults in the United States living with some form of CHD [9]. Specifically, this population has been shown to have an association with increased cardiovascular (CV) disease risk into adulthood such as stroke, heart failure, and coronary artery disease [10]. These patients can experience medical and surgical interventions in childhood and adolescence that may contribute to this increased risk factor profile [11, 12]. As the adult CHD population continues to expand, these patients are at higher risk than the general population for inherent and acquired CV disease. Elevated HbA1c, as a measure of cardiometabolic health, has been shown to be a significant, modifiable risk factor for adverse CV outcomes and overall mortality. Earlier HbA1c screening and glycemic control interventions have been shown to reduce acquired CV disease [13, 14]. The clinical significance of HbA1c screening in the pediatric CHD population has thus far been under-reported.

There is limited data on the rates of HbA1C screening in the CHD population. Furthermore, the prevalence of elevated HbA1c and correlation with other CV risk factors in children with CHD have not been previously described. Addressing this research gap could improve understanding of the cardiometabolic risk profile in this population that can be screened for and managed to improve long term CV outcomes. Our study primarily focused on evaluating HbA1c screening rates in children with CHD. We also sought to better describe the characteristics of youth with elevated HbA1c and CHD.

## Methods

### Study Design, Study Population and Data Acquisition

We conducted a retrospective study of children with CHD under 18 years at Nationwide Children’s Hospital (Columbus, OH) that were included in our clinical echocardiogram database from the time of adoption of the current electronic health record system (2012) up to the time that our division transitioned to a new echocardiographic reading software program (2019). Our clinical echocardiogram database was used from 2003 to 2019 and stored every echocardiogram done at our center for patients with and without CHD. A subset of the clinical echocardiogram database was formed to focus on echocardiograms from 2012 to 2019, as more robust data was available with our current electronic health record system, with the initial purpose of this subset database to evaluate blood pressure and left ventricular echocardiogram parameters in patients with CHD [15]. This subset database was subsequently utilized for our study. All individuals receiving an echocardiogram between 2012 and 2019 were included in the database except for patients with hypoplastic left heart syndrome or other cardiac conditions where left ventricular parameters were not available. Children without CHD were excluded from the analysis. Data acquired included the following: lipid panel, HbA1c values, demographic characteristics (age, sex, race), vital signs (height, weight, systolic/diastolic blood pressures) taken at time of echocardiogram, and CHD lesion characteristics. Race was included given the ADA screening criteria including race as a risk factor to determine screening eligibility. The database assigned race by patient reported demographics in the electronic health record. This study was approved by our Institutional Review Board.

Since a primary aim of the analysis was identifying potential disparities by examining differences in demographic and vital sign values by cohorts, we included only those with complete information on age, sex, race, height, weight, systolic blood pressure, and diastolic blood pressure. Data elements outside of biologically plausible ranges set by investigators were also dropped (Supplemental Table 3). There were a total of 6529 patients aged < 18 years with CHD and echocardiograms with information on left ventricular dimensions between 2003 and 2019. After excluding patients with missing data and matching to the start of our current electronic health record system in 2012, there were 2764 patients in the final analysis data set. The study population was divided and compared between groups who had a HbA1c level drawn during the study period and those without HbA1c levels. Following this, an additional stratification was made for comparison between those with normal versus elevated HbA1c values. We then evaluated the entire cohort for screening eligibility for diabetes and metabolic syndrome by a subset of ADA [6] and the International Diabetes Federation (IDF) criteria [16].

### Relevant Criteria

The following criteria were implemented to create composite variables during the data analysis. These data elements include a subset of the full ADA and IDF criteria and were limited to available data:

- **Criteria for pre-diabetes/diabetes by HbA1c**:
  - Normal, < 5.7%.
  - Prediabetes, 5.7% ≤ HbA1c < 6.5%.
  - Diabetes ≥ 6.5%.
- **Criteria for dyslipidemia by lipid panel**:
  - Total cholesterol ≥ 200 mg/dL.
  - Low-density lipoprotein cholesterol [LDL-C] ≥ 130 mg/dL.
  - Triglycerides ≥ 130 mg/dL.
  - High-density lipoprotein cholesterol [HDL-C] < 40 mg/dL.
  - Non-HDL ≥ 145 mg/dL.
- **ADA criteria for diabetes screening**:
  - Patients age ≥ 10 years with overweight or obesity (BMI ≥ 85^th^ percentile) who have at least one more risk factor.
    - Race: American Indian, African American, Latino, Asian American, or Pacific Islander race or ethnicity.
    - Hypertension.
    - Dyslipidemia.
- **IDF criteria for metabolic syndrome**:
  - ≥ 2 factors of:
    - Increased waist circumference (used obesity BMI ≥ 95th percentile as proxy).
    - Dyslipidemia.
    - Hypertension.
- **CDC childhood obesity criteria**:
  - Overweight: 85th percentile ≤ BMI < 95th percentile.
  - Obese: BMI ≥ 95th percentile.
- **Pediatric hypertension criteria**:
  - < 13 years (using percentiles).
    - Normal: < 90th percentile.
    - Elevated: 90th percentile ≤ SBP < 95th percentile.
    - Stage I: 95th percentile ≤ SBP < 95th percentile + 12 mmHg.
    - Stage II: SBP ≥ 95th percentile + 12 mmHg.
  - ≥ 13 years (using adult cutoff).
    - Normal: SBP < 120 mmHg.
    - Elevated: 120 ≤ SBP < 130 mmHg.
    - Stage I: 130 ≤ SBP < 140 mmHg.
    - Stage II: SBP ≥ 140 mmHg.

Fasting or non-fasting status for the lipid panels was not available so we also used an alternative criteria of HDL-C < 40 mg/dL and/or non-HDL-C ≥ 145 mg/dL to define dyslipidemia as per American Academy of Pediatrics recommendations for interpreting non-fasting lipid panels [17].

### CHD Lesion Characteristics

For patients that received HbA1c values, CHD lesions were classified according to the following categories: tetralogy of Fallot (TOF), transposition of the great arteries (TGA), truncus arteriosus (TA), left-to-right shunts (atrial septal defect, ventricular septal defect, atrioventricular canal defect, patent ductus arteriosus), pulmonic stenosis (PS), any level of left ventricular obstruction through the left ventricular outflow tract (LVOT) or aorta were classified in the LVOT group (e.g., subvalvular aortic stenosis, valvar stenosis, supravalvular stenosis, coarctation of the aorta). Minor and uncommon lesions were included in a separate “other” category that included aortic valve aneurysm, tricuspid valve prolapse, mitral valve prolapse, pulmonary vein disease, mitral valve regurgitation, aortic root repair, coronary anomalies, patent foramen ovale (PFO), and heart transplants. Separate categories were made for bicuspid valves without stenosis and for single ventricle lesions with analysis being focused on those patients with a systemic left ventricle. Screening data and rates of elevated HbA1c values were then grouped according to CHD lesion categories.

### Statistical Analysis

Demographic and anthropometric characteristics were summarized using median (interquartile range) for continuous variables and frequency (percent) for categorical variables. Rates of screening eligibility and prevalence of metabolic syndrome between those with and without HbA1c labs performed were compared using Pearson’s Chi-squared test. Differences in characteristics between comparison groups were quantified as either difference in value for continuous variables or difference in observed percent for categorical variables. Bootstrap resampling with 200 replicates was utilized to generate 95% confidence intervals for difference in values or percent. Bootstrap confidence intervals are used as measures of variability and were not meant to be utilized as inferential tests.

## Results

Demographics and vital signs for all patients included with complete information are incorporated into Table 1. Out of 2764 total patients included, 219 (7.9%) had HbA1c lab performed. Overall, the majority of patients were White (81.3%) with the second most common race being Black (12.9%). In the cohort with HbA1c lab values, there was an increased proportion of Black patients (23.3% vs. 12.0%). Median systolic and diastolic blood pressures in the overall cohort were not elevated. However, a larger proportion of patients in the HbA1c screening group had blood pressure categories that were in the elevated (18.7% vs. 14.1%), Stage I (16.4% vs. 12.8%), or Stage II categories (5.5% vs. 4.4%). The HbA1c group had higher BMI percentiles with the median range being in the overweight category at 0.92 and higher proportion of those overweight or obese. There was a further shift in the HbA1c group into the overweight (20.1% vs. 13.9%) and obese (40.2% vs. 12.1%) categories. A high proportion of patients screened with a HbA1c level also had a screening lipid panel (67.1%), whereas only 4.6% of those without a HbA1c had a screening lipid panel. Of those in the HbA1c group with lipid panels, 66% (97/147) were dyslipidemic whereas 50% (58/116) of those with lipid panels in the non-HbA1c group were dyslipidemic.

**Table 1:** Demographics and vitals by HbA1c lab availability

Characteristic	Had HbA1c lab performed?		
	Overall, *N* = 2764^a^	No, *N* = 2545^a^	Yes, *N* = 219^a^
Age (years)	10.0 (6.2, 13.9)	9.9 (6.0, 13.9)	10.7 (8.0, 14.3)
Sex			
Male	1506 (54.5%)	1396 (54.9%)	110 (50.2%)
Female	1258 (45.5%)	1149 (45.1%)	109 (49.8%)
Race			
White	2247 (81.3%)	2092 (82.2%)	155 (70.8%)
Black	357 (12.9%)	306 (12.0%)	51 (23.3%)
Asian	98 (3.5%)	95 (3.7%)	3 (1.4%)
Native American	38 (1.4%)	32 (1.3%)	6 (2.7%)
Hispanic	18 (0.7%)	14 (0.6%)	4 (1.8%)
Other	6 (0.2%)	6 (0.2%)	0 (0.0%)
Systolic blood pressure	108 (99, 118)	108 (99, 118)	110 (102, 120)
Diastolic blood pressure	60 (55, 67)	60 (55, 67)	62 (56, 69)
Systolic blood pressure percentile	0.72 (0.46, 0.90)	0.71 (0.46, 0.90)	0.83 (0.57, 0.94)
Diastolic blood pressure percentile	0.57 (0.37, 0.77)	0.57 (0.37, 0.77)	0.60 (0.39, 0.79)
Blood pressure category			
Normal	1,877 (67.9%)	1,747 (68.6%)	130 (59.4%)
Elevated	400 (14.5%)	359 (14.1%)	41 (18.7%)
Stage I	363 (13.1%)	327 (12.8%)	36 (16.4%)
Stage II	124 (4.5%)	112 (4.4%)	12 (5.5%)
BMI percentile	0.62 (0.31, 0.89)	0.60 (0.28, 0.86)	0.92 (0.65, 0.98)
BMI category			
Normal weight	1,971 (71.3%)	1,884 (74.0%)	87 (39.7%)
Overweight	398 (14.4%)	354 (13.9%)	44 (20.1%)
Obese	395 (14.3%)	307 (12.1%)	88 (40.2%)
Had lipid panel performed?	263 (9.5%)	116 (4.6%)	147 (67.1%)
Dyslipidemic?	155 (5.6%)	58 (2.3%)	97 (44.3%)
Dyslipidemic by HDL-C/Non-HDL-C	130 (4.7%)	48 (1.9%)	82 (37.4%)

*BMI* body mass index, *HbA1c* hemoglobin A1c, *HDL-C* high density lipoprotein cholesterol

^a^Median (IQR); n (%)

**Fig. 1:**
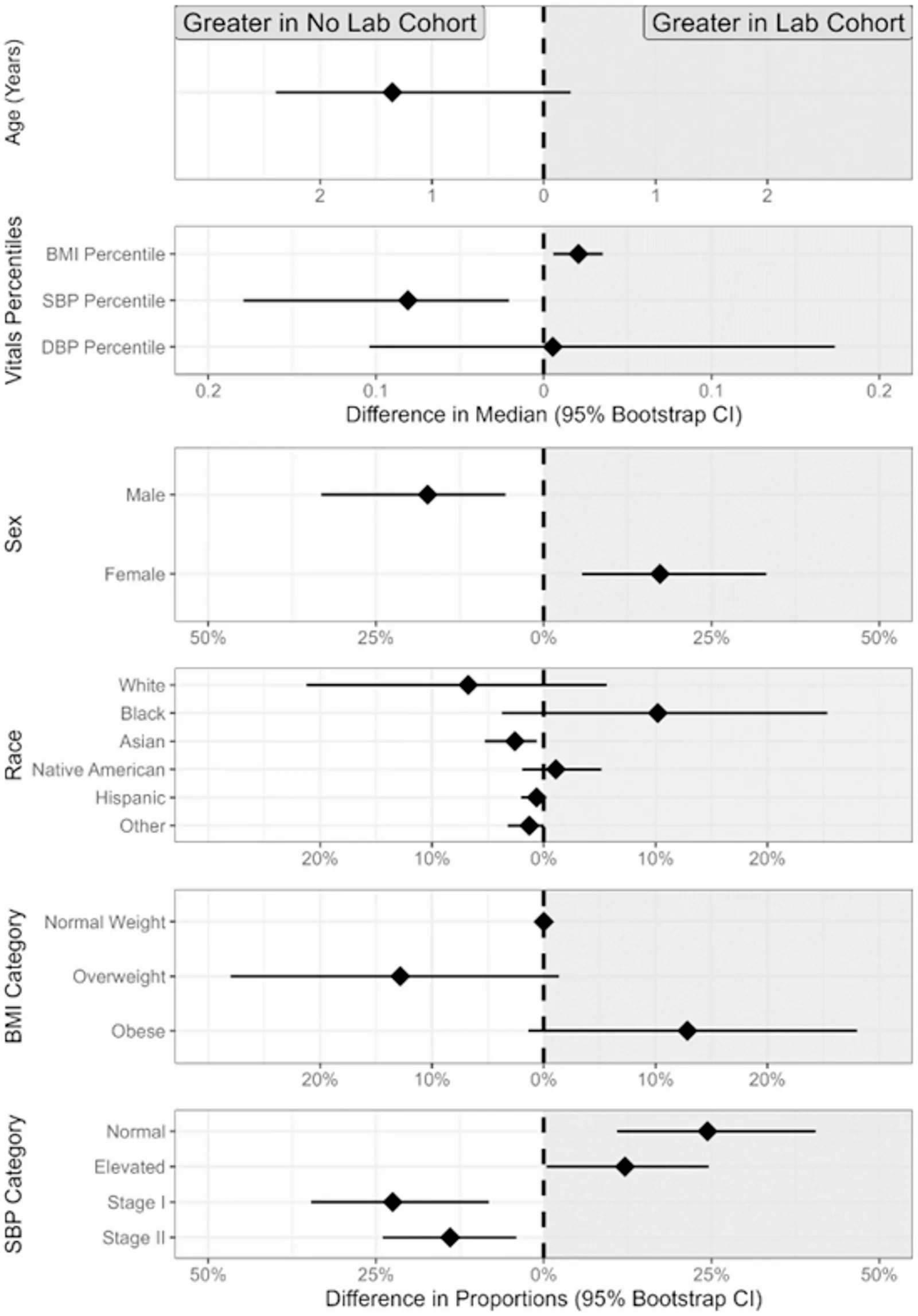
Differences among screen eligibility by HbA1c availability. Differences in age, vitals percentile, sex, race, body mass index (BMI) category, and systolic blood pressure (SBP) percentiles were evaluated

Table 2; Fig. 1 compare characteristics of ADA screen-eligible individuals by if an HbA1c lab value was obtained and provide observed differences between groups. There were 213 individuals who were ADA screen-eligible, but only 58 of them had HbA1c levels drawn. There was a significantly higher proportion of Black individuals in the lab group compared to the screen-eligible group without labs. The lab group had a significantly higher proportion of individuals in the obese category. Interestingly, the no lab group had a significantly higher proportion of individuals in the Stage I and Stage II blood pressure categories compared to the lab group. Overall, of those who were screen eligible and had labs available (*n* = 58), 62% (36/58) had HbA1c levels in the normal range and 38% (22/58) had HbA1c levels in the prediabetic or diabetic range. Figure 1 provides a visual representation of the differences observed in Table 2.

**Table 2:** Differences between screen eligible individuals by HbA1c availability

Characteristic	Had HbA1c lab performed given screen-eligible by ADA?		Difference (95% CI)
	No, *N* = 155^a^	Yes, *N* = 58^a^	
Age (years)	14.6 (12.7, 16.2)	13.2 (11.8, 15.3)	1.36 (− 0.24, 2.4)
Sex			
Male	99 (63.9%)	27 (46.6%)	0.17 (0.06, 0.33)
Female	55 (36.1%)	31 (53.4%)	− 0.17 (− 0.33, − 0.06)
Race			
White	104 (67.1%)	35 (60.3%)	0.07 (− 0.06, 0.21)
Black	43 (27.7%)	22 (37.9%)	− 0.1 (− 0.25, 0.04)
Asian	4 (2.6%)	0 (0.0%)	0.03 (0.01, 0.05)
Native American	1 (0.6%)	1 (1.7%)	− 0.01 (− 0.05, 0.02)
Hispanic	1 (0.6%)	0 (0.0%)	0.01 (0, 0.02)
Other	2 (1.3%)	0 (0.0%)	0.01 (0, 0.03)
BMI percentile	0.96 (0.92, 0.98)	0.98 (0.94, 0.99)	− 0.02 (− 0.04, − 0.01)
BMI category			
Normal weight	0 (0.0%)	0 (0%)	0 (0, 0)
Overweight	68 (43.9%)	18 (31.0%)	0.13 (− 0.01, 0.28)
Obese	87 (56.1%)	40 (69.0%)	− 0.13 (− 0.28, 0.01)
Systolic blood pressure percentile	0.97 (0.88, 0.99)	0.89 (0.62, 0.98)	0.08 (0.02, 0.18)
Diastolic blood pressure percentile	0.57 (0.37, 0.77)	0.57 (0.37, 0.75)	− 0.01 (− 0.17, 0.1)
Blood pressure category			
Normal	29 (18.7%)	25 (43.1%)	− 0.24 (− 0.4, − 0.11)
Elevated	16 (10.3%)	13 (22.4%)	− 0.12 (− 0.26, − 0.02)
Stage I	75 (48.4%)	15 (25.9%)	0.23 (0.08, 0.35)
Stage II	35 (22.6%)	5 (8.6%)	0.14 (0.04, 0.24)

*ADA* American Diabetes Association, *BMI* body mass index, *HbA1c* hemoglobin A1c

^a^Median (IQR); n (%)

Table 3 depicts the prevalence of ADA diabetes screening eligibility and IDF metabolic syndrome screening eligibility compared between those with and without an HbA1c lab performed. In the HbA1c group, 26.5% of the patients met criteria for screening as compared to 6.1% of the group without an HbA1c value (*p* < 0.001). The HbA1c group also had 31.1% meet criteria for metabolic syndrome screening compared to 4.8% of the group without an HbA1c value (*p* < 0.001). Of the entire cohort with an HbA1c value obtained, 16.4% had a value in the prediabetic range and 5.5% had a value in the diabetic range with median (IQR) HbA1c of 5.3 (5.1, 5.6) %.

**Table 3:** Prevalence of screen eligibility and metabolic syndromes by HbA1c lab availability

Characteristic	Had HbA1c lab performed			p value^b^
	Overall, *N* = 2764^a^	No, *N* = 2545^a^	Yes, *N* = 219^a^	
Meets ADA diabetes screening guidelines	213 (7.7%)	155 (6.1%)	58 (26.5%)	< 0.001
Meets IDF guidelines for metabolic syndrome	191 (6.9%)	123 (4.8%)	68 (31.1%)	< 0.001
Diabetes by HbA1c				–
Normal	2716 (98.3%)	–	171 (78.1%)	
Prediabetes	36 (1.3%)	–	36 (16.4%)	
Diabetes	12 (0.4%)	–	12 (5.5%)	

*ADA* American Diabetes Association, *HbA1c*, hemoglobin A1c, *IDF* International Diabetes Federation

^a^n (%)

^b^Pearson’s Chi-squared test

The study population that had an HbA1c value obtained was further stratified into CHD lesion type with comparisons made between HbA1c levels, if ADA diabetes screening criteria were met, and if IDF metabolic syndrome criteria were met (Supplemental Table 1). Across all lesion types there was a sizable proportion of patients with elevated HbA1c values in the prediabetic and diabetic ranges. The lesions with the most patients in this cohort included left-to-right shunts (*n* = 75), Minor/other CHD (*n* = 45), and LVOT obstructions (*n* = 33). The lesion with the highest percentage of patients in the diabetic range was bicuspid aortic valve (BAV) without stenosis (11%).

Supplemental Table 2 compares those with normal HbA1c levels and those with elevated HbA1c levels. As previously mentioned, 219 individuals had a lab drawn with 48 (21.9%) having values in the elevated range. The groups had similar characteristics with the main difference being a higher age in the cohort with elevated values. BMI categories, systolic and diastolic blood pressure percentiles, and blood pressure categories were not significantly different between the groups.

## Discussion

In the CHD population, there is an inherent risk of increased CV disease that may be exacerbated by multiple coinciding risk factors such as elevated HbA1c and dyslipidemia. These risk factors are potentially modifiable and may progress throughout life. With earlier screening and disease modifying behaviors and therapies, there is the potential to mitigate this risk. Screening and incidence rates of prediabetes and diabetes mellitus in pediatric CHD patients have thus far been unreported and represent a possibly significant gap in care. Our retrospective study reports HbA1c screening rates and associated cardiometabolic risk factors in a large population of CHD patients.

### Overall Screening Rates

In our study population with CHD patients, 7.7% of the total cohort met ADA screening eligibility guidelines. This is a smaller percentage compared to that found by Wallace et al. where around 25.5% of US children and adolescents fall into the screen-eligible category [7]. Of those who were identified as being screen eligible in our study, only 27.2% (58/213) had a lab performed. It is likely that the number of children with CHD meeting ADA screening criteria was underestimated by our study, partially due to inability to evaluate for some of the risk factors such as family history or maternal history of gestational diabetes. Furthermore, many patients did not have a lipid panel obtained. Some of these patients could have dyslipidemia, which would qualify them for diabetes screening and metabolic syndrome. This reveals a significant gap in care for this population with current practices not meeting national guideline standards with screening rates well below expected practice. This gap in screening demonstrates a lapse in opportunity to identify and address an additional, modifiable risk factor in the CHD population.

### Youth with Elevated HbA1c

HbA1c value is defined as normal with a value of < 5.7% with higher values being categorized in the pre-diabetic or diabetic ranges. Prior studies have suggested that while many patients may be eligible for screening, a small number of those tested in the screening-eligible population, around 4%, will meet criteria for HbA1c defined hyperglycemia [7]. In our study population, 219 patients had a lab value obtained and 48 (21.9%) of these patients had values in either the prediabetic (*N* = 36 ) or diabetic range (*N* = 12). Of those 58 patients who were screen eligible and had labs available, there was a large proportion (38%; 17 prediabetic, 5 diabetic) of patients with prediabetic or diabetic range HbA1c values. Given the limitations of our available data, our study was not designed to determine the true prevalence of elevated HbA1c in this patient population. However, it is notable that those who were screen-eligible and actually underwent screening had a sizable proportion meeting prediabetes/diabetes criteria. This finding supports increased screening of this patient population using the available screening criteria.

The current broader ADA screening criteria pertain to the general population of asymptomatic children. Based on our findings, the ADA criteria also appears to be helpful in youth with CHD to identify individuals with potential risk for prediabetes and diabetes. Considering prior evidence that links prediabetes to increased CV risk both in children and in adults, this cohort of CHD patients who are accumulating multiple coinciding CV risk factors over time may need our heightened attention [18, 19]. This need is further demonstrated by our findings that among children who were not eligible per ADA screening criteria but did have an A1c drawn (*N* = 161), 16% of these patients (*N* = 26) had HbA1c values in the prediabetic or diabetic range.

### Coinciding Risk Factors

Prior studies have depicted the implications of childhood risk factors on predicting adult CV disease. Certain CV risk factors including BMI, SBP, and total cholesterol levels that emerged in part during childhood were linked to CV events and death in adulthood [20]. Elevated HbA1c is one of many cardiometabolic risk factors in our patient population that was evaluated. Several other risk factors were obtained that coincide to produce potentially cumulative CV risk. The group with HbA1c values had a higher shift into the overweight and obese categories while also being more likely to be dyslipidemic. Additionally, we noted a high proportion of patients (40.5%) in this cohort having elevated blood pressure. This is supported by the fact that 31% of this cohort met IDF guidelines for metabolic syndrome. While these risk factors are well known in the general population, this study demonstrates multiple specific findings in the pediatric CHD population that demonstrate accumulating CV risk in an already predisposed population.

### Limitations

While our study is one of the largest to evaluate and characterize CHD patients with HbA1c screening data, it has several limitations. Our study is a single center retrospective cross-sectional study which inherently limits the generalizability of our findings across different institutions and areas of the country. Moreover, our data comes from a dataset whose initial purpose was to evaluate blood pressures and rates of left ventricular hypertrophy in the pediatric CHD population. This parameter constrained us to look at CHD lesions with a systemic left ventricle, notably leaving out any hypoplastic left heart syndrome (HLHS) patients. Patient medications were not available in this database. For example, this may explain the findings of lower BP trend in the screen eligible cohort (Table 2) as potentially being the result of intervention including medical treatment leading to confounding bias. We used a subset of ADA and IDF criteria to identify screen-eligible patients based on our available data. Using the full criteria could have possibly increased the total number of screen-eligible patients. It was not specified in the database whether the lab data was obtained at the cardiology encounter and thus screening for metabolic syndrome could have been performed by providers other than cardiologists. Additionally, only HbA1c values were investigated as a diagnostic measure for pre-diabetes and diabetes diagnosis, and other measures such as fasting plasma glucose were not evaluated. Finally, it is possible that labs were obtained for the patients outside of our health system and therefore true screening rates might have been higher. This issue highlights potential gaps of awareness as to which patients should be screened for diabetes.

## Conclusion

Despite these limitations, this study offers a novel insight into rates of screening and metabolic risk profile in the CHD population. We found that screening rates for prediabetes and diabetes are low in screen-eligible youth with CHD. This is important as it demonstrates an implementation gap between recommendations and clinical practice. Our findings of high rates of prediabetes and diabetes in screen-eligible children with CHD suggest the need for improved screening practices in this patient population. Having elevated HbA1c is an independent and modifiable risk factor for adverse CV outcomes, and if at-risk individuals are identified, interventions can be taken to affect this modifiable risk factor. Future prospective studies are needed to identify metabolic risk factors in children with CHD compared to the general population. These future analyses in particular should call attention to the link between standard CV risk factors and social drivers of health, taking into consideration one’s underlying CHD. Determination of barriers and facilitators to screening and longitudinal assessment of children with CHD for cardiometabolic risk factors could inform interventions to address these risks.

## Supplementary Information

Below is the link to the electronic supplementary material.

Supplementary Material 1

## Data Availability

The data that support the findings of this study are not openly available but are available from the corresponding author upon reasonable request. Data are located in controlled access data storage at Nationwide Children’s Hospital (Columbus, OH).

## Abbreviations

HbA1c: Hemoglobin A1c
CHD: Congenital heart disease
CV: Cardiovascular
ADA: American Diabetes Association
BP: Blood pressure
BMI: Body mass index
