# Supplementary material for: Hemoglobin A1c Screening Practices and Characteristics in Youth with Congenital Heart Disease
Source: Pediatr Cardiol. 2025 Nov 6;47(7):3060–8. doi: 10.1007/s00246-025-04092-0 (PMC13597541; doi:10.1007/s00246-025-04092-0)
Supplement: Supplementary file 1 [file 246_2025_4092_MOESM1_ESM.docx]

***Supplementary Information***

**Article Title:** Hemoglobin A1c Screening Practices and Characteristics in Youth with Congenital Heart Disease

**Journal:** Pediatric Cardiology

**Authors:**

Jonathan A. Wheeler^1^, MD; Phillip S. Cheng^2^, MD; Chance Alvarado, MS^3,4,5^; Leena Mamilly, MD ^6^; Aaron Walsh, MD^7^; Kan N. Hor, MD^2,3*^; Andrew H. Tran, MD, MPH, MS^2,3*^

^*^*Contributed equally as co-senior authors*

**Affiliation(s):**

^1^Department of Pediatrics, Division of Pediatric Cardiology, Baylor College of Medicine, Texas Children's Hospital, Houston, Texas.

^2^Department of Pediatrics, The Ohio State University, Columbus, OH, USA

^3^The Heart Center, Nationwide Children’s Hospital, Columbus, OH, USA

^4^Biostatistics Resource, Abigail Wexner Research Institute, Nationwide Children's Hospital, Columbus, OH, 43205, USA.

^5^Center for Biostatistics, The Ohio State University, Wexner Medical Center, Columbus, OH, 43210, USA.

^6^Section of Endocrinology, Department of Pediatrics, Nationwide Children's Hospital/The Ohio State University College of Medicine, Columbus, Ohio, USA

^7^The Heart Institute, Le Bonheur Children’s Hospital, Memphis, TN, USA

**Abbreviations:** Hemoglobin A1c (HbA1c), Congenital Heart Disease (CHD), Cardiovascular (CV), American Diabetes Association (ADA), Blood Pressure (BP), Body Mass Index (BMI)

**Corresponding author:**

Andrew H. Tran, MD, MPH, MS

The Heart Center, Nationwide Children’s Hospital

700 Childrens Drive, Columbus, OH 43205

Andrew.Tran@nationwidechildrens.org

ORCID: 0000-0001-5777-4611

**Supplemental Table 1:** Prevalence of Screen Eligibility and Metabolic Syndromes by CHD Lesion Type

|  | **CHD Lesion Type** | | | | | | | | | |
| --- | --- | --- | --- | --- | --- | --- | --- | --- | --- | --- |
| **Characteristic** | **TOF**, N = 20^1^ | **TGA**, N = 10^1^ | **Truncus**, N = 1^1^ | **Left to Right Shunts**, N = 75^1^ | **PS**, N = 14^1^ | **LVOT**, N = 33^1^ | **BAV without Stenosis**, N = 18^1^ | **Single Ventricle CHD - Hypoplastic RV**, N = 3^1^ | **Minor/Other CHD**, N = 45^1^ |  |
| **HbA1c (%)** | 5.3  (5.1, 5.6) | 5.4  (5.3, 5.6) | 5.9  (5.9, 5.9) | 5.3  (5.0, 5.6) | 5.4  (5.2, 5.6) | 5.2  (5.0, 5.6) | 5.5  (5.1, 5.9) | 5.6  (5.6, 5.6) | 5.3  (5.1, 5.6) |  |
| **Meets ADA Diabetes Screening Guidelines** | 4  (20%) | 3  (30%) | 0  (0%) | 23 (31%) | 5  (36%) | 9  (27%) | 5  (28%) | 0  (0%) | 9  (20%) |  |
| **Meets IDF Guidelines for Metabolic Syndrome** | 6  (30%) | 2  (20%) | 1 (100%) | 24 (32%) | 7  (50%) | 11  (33%) | 8  (44%) | 0  (0%) | 9  (20%) |  |
| **Diabetic by HbA1c?** |  |  |  |  |  |  |  |  |  |  |
| Normal | 16 (80%) | 9 (90%) | 0  (0%) | 59  (79%) | 11  (79%) | 27  (82%) | 12  (67%) | 3  (100%) | 34 (76%) |  |
| Prediabetes | 3 (15%) | 1 (10%) | 1 (100%) | 12 (16%) | 3 (21%) | 3 (9%) | 4 (22%) | 0  (0%) | 9 (20%) |  |
| Diabetes | 1 (5%) | 0  (0%) | 0  (0%) | 4 (5%) | 0 (0%) | 3 (9%) | 2 (11%) | 0  (0%) | 2 (4%) |  |
| ^1^Median (IQR); n (%) | | | | | | | | | | |

ADA, American Diabetes Association; BAV, bicuspid aortic valve; CHD, congenital heart disease; HbA1c, hemoglobin A1c; IDF, International Diabetes Federation; LVOT, left ventricular outflow tract; PS, pulmonary stenosis; RV, right ventricle; TGA, transposition of the great arteries; TOF, Tetralogy of Fallot

**Supplemental Table 2:** Differences among Those with Elevated and Normal HbA1c Levels

|  | **Elevated HbA1c Given Had HbA1c Lab?** | |  |  |
| --- | --- | --- | --- | --- |
| **Characteristic** | **No**, N = 171^1^ | **Yes**, N = 48^1^ | **Difference (95% CI)** | |
| **Age (Years)** | 10.5 (7.6, 14.3) | 11.8 (8.7, 14.1) | -1.34 (-2.77, 1.45) | |
| **Sex** |  |  |  | |
| Male | 84 (49.1%) | 26 (54.2%) | -0.05 (-0.21, 0.11) | |
| Female | 87 (50.9%) | 22 (45.8%) | 0.05 (-0.11, 0.21) | |
| **Race** |  |  |  | |
| White | 124 (72.5%) | 31 (64.6%) | 0.08 (-0.07, 0.21) | |
| Black | 36 (21.1%) | 15 (31.3%) | -0.1 (-0.24, 0.03) | |
| Asian | 3 (1.8%) | 0 (0.0%) | 0.02 (0, 0.04) | |
| Native American | 5 (2.9%) | 1 (2.1%) | 0.01 (-0.04, 0.05) | |
| Hispanic | 3 (1.8%) | 1 (2.1%) | 0 (-0.06, 0.03) | |
| Other | 0 (0.0%) | 0 (0.0%) | 0 (0, 0) | |
| **BMI Percentile** | 0.90 (0.64, 0.98) | 0.96 (0.82, 0.99) | -0.05 (-0.11, 0.01) | |
| **BMI Category** |  |  |  | |
| Normal Weight | 73 (42.7%) | 14 (29.2%) | 0.14 (-0.01, 0.28) | |
| Overweight | 34 (19.9%) | 10 (20.8%) | -0.01 (-0.15, 0.11) | |
| Obese | 64 (37.4%) | 24 (50.0%) | -0.13 (-0.3, 0.03) | |
| **Systolic Blood Pressure Percentile** | 0.83 (0.57, 0.94) | 0.85 (0.58, 0.94) | -0.03 (-0.12, 0.15) | |
| **Diastolic Blood Pressure Percentile** | 0.60 (0.39, 0.81) | 0.57 (0.46, 0.77) | 0.03 (-0.09, 0.11) | |
| **Blood Pressure Category** |  |  |  | |
| Normal | 103 (60.2%) | 27 (56.3%) | 0.04 (-0.1, 0.2) | |
| Elevated | 33 (19.3%) | 8 (16.7%) | 0.03 (-0.09, 0.14) | |
| Stage I | 25 (14.6%) | 11 (22.9%) | -0.08 (-0.21, 0.05) | |
| Stage II | 10 (5.8%) | 2 (4.2%) | 0.02 (-0.08, 0.08) | |

^1^Median (IQR); n (%)

BMI, body mass index; HbA1c, hemoglobin A1c

**Supplemental Table 3.** Data Validation Ranges

| **Parameter** | **Validation Range** |
| --- | --- |
| Height | <243 cm |
| BMI | 10 < BMI ≤ 100 kg/m^2^ |
| Systolic blood pressure | > 50 mmHg |
| Diastolic blood pressure | > 25 mmHg |
| HDL-C | > 10 mg/dL |
| Triglycerides | > 40 mg/dL |

BMI, body mass index; HDL-C, high density lipoprotein cholesterol
